# Global analysis of protein lysine lactylation profiles in the marine bacterium *Photobacterium damselae* subsp. *damselae*

**DOI:** 10.3389/fmicb.2025.1539893

**Published:** 2025-06-17

**Authors:** Yongxiang Yu, Haozhe Liu, Chunyuan Wang, Yingeng Wang, Xiaojun Rong, Meijie Liao, Bin Li, Xingling Yi, Zheng Zhang

**Affiliations:** ^1^State Key Laboratory of Mariculture Biobreeding and Sustainable Goods, Yellow Sea Fisheries Research Institute, Chinese Academy of Fishery Sciences, Qingdao, Shandong, China; ^2^Laboratory for Marine Fisheries Science and Food Production Processes, Laoshan Laboratory, Qingdao, Shandong, China; ^3^Weimi Biotechnology Co., Ltd., Hangzhou, Zhejiang, China

**Keywords:** *Photobacterium damselae* subsp. *damselae*, post-translational modification (PTM), lysine lactylation (Klac), proteomics, protein-protein interaction (PPI) network

## Abstract

Lysine lactylation (K_*lac*_) is a recently discovered post-translational modification (PTM) widespread across species, playing a crucial role in cellular processes and associated with pathological conditions. *Photobacterium damselae* subsp. *damselae*, a marine bacterium within the Vibrionaceae family, is a notable pathogen in aquaculture, offering a valuable model for investigating the evolution of pathogenicity from environmental ancestors and assessing the impact of genetic diversity-generating mechanisms on bacterial populations. Therefore, we conducted the first systematic analysis of K_*lac*_ modification in *P. damselae* using highly sensitive proteomic techniques. A total of 1,352 K_*lac*_ modification sites were identified on 486 proteins. The analysis of GO annotations and KEGG pathways for the identified K_*lac*_-modified proteins revealed their widespread distribution in subcellular compartments, indicating their involvement in diverse cellular functions and metabolic pathways, particularly in ribosome and protein biosynthesis, as well as central carbon metabolism. Furthermore, 20 highly connected K_*lac*_ protein clusters were extracted from the global protein-protein interaction (PPI) network, indicating that K_*lac*_ modification tends to occur on proteins associated with specific functional clusters. These findings enhance our understanding of the functional role of K_*lac*_ modification and provide a dataset for further exploration of its impact on the physiology and biology of *P. damselae*.

## Introduction

*Photobacterium damselae* subsp. *damselae*, formerly designated as *Vibrio damsela*, is a marine bacterium affiliated with the Vibrionaceae family. Nonetheless, based on DNA-DNA hybridization data and 16S rRNA sequence analysis, it has been taxonomically categorized within the species *P. damselae*, coexisting with another fish bacterial pathogen, *P. damselae* subsp. *piscicida* (formerly identified as *Pasteurella piscicida*) (Gauthier et al., 1995). It is currently recognized as an important bacterial pathogen in marine aquaculture, similar to various Vibrio species such as *V. parahaemolyticus*, and has been detected in various aquaculture species in different countries and regions (Osorio et al., 2018). Moreover, due to the ability of most of *P. damselae* subs. *damselae* strains to thrive at 37°C (a temperature inhibitory for subsp. *piscicida*), there is a potential for parasitism and infection in homoiothermic animals (Rivas et al., 2013), potentially culminating in opportunistic infections in humans and evolving into fatal necrotizing fasciitis (Rivas et al., 2013; Yamane et al., 2004). Presently, the virulence factors and pathogenic mechanisms of the bacterium remain incompletely elucidated; however, certain virulence factors have been reported and reviewed in two papers (Dong et al., 2022; Li et al., 2023). The authors of one of the two papers, Osorio et al. (2018), proposed that *P. damselae* subs. *damselae*, together with other pathogenic members of the Vibrionaceae family, constitutes an excellent model system for exploring the evolution of pathogenicity from environmental ancestors and the impact of genetic diversity-generating mechanisms on bacterial populations. Despite the shared environmental similarities among all Vibrionaceae bacteria, their dizzying diversity in pathobiology and adaptive gene functions results in each species within the Vibrionaceae family exhibiting its distinctive biological strategy. *P. damselae* subs. *damselae* is no exception, and, in fact, it represents a fascinating biological model, the elucidation of whose adaptive molecular mechanisms of biology and pathogenesis is undoubtedly warranted. Consequently, performing proteomic analysis of post-translational modifications in *P. damselae* subs. *damselae* will contribute to unveiling its physiological processes.

Protein post-translational modification (PTM), which involves the dynamic and reversible modification of proteins, is considered one of the most effective strategies for altering protein properties, expanding the functional diversity of existing proteins, and enhancing the cellular control of various biological processes and metabolic pathways (Pan et al., 2015). Extensive research on PTMs in both eukaryotic and prokaryotic cells has consistently identified lysine residues in proteins as the most prevalent targets for modification. In recent years, there has been extensive research on lysine modifications in both eukaryotic and prokaryotic cells, with acylation modifications of lysine being a widely identified form of modification. These modifications include methylation (K_*me*_), acetylation (K_*ac*_), crotonylation (K_*cr*_), butyrylation (K_*bu*_), glutarylation (K_*glu*_), succinylation (K_*succ*_), malonylation (K_*mal*_), propionylation (K_*pr*_), ubiquitination (K_*ub*_), 2-hydroxyisobutyrylation (K_*hib*_) (Pan et al., 2015), and lactylation (K_*lac*_ or K_*la*_), the latter being a recent discovery made 5 years ago (Zhang et al., 2019).

Recent data indicate that lactate functions not only as a readily accessible fuel and a metabolic buffer bridging glycolysis and oxidative phosphorylation within cells and intracellular compartments, but also as a multifunctional signaling molecule that acts as a coordinator of whole-body metabolism. Lactate can induce various biological effects, such as reducing fat breakdown, modulating the immune system, exerting anti-inflammatory effects, promoting wound healing, and enhancing exercise performance in association with gut microbiota (Lee, 2021). Therefore, exploring protein lactylation not only contributes to elucidating the physiological functions of lactate but, more importantly, by revealing the functions and classifications of proteins modified by lactylation, it facilitates a deeper understanding of the potential regulatory roles of lactylation in physiological functions.

Lactylation at lysine residue (K_*lac*_) modification is a novel PTM first discovered on histones in mammalian cells in 2019 (Zhang et al., 2019). It has been observed that K_*lac*_ modification on histones modulates macrophage polarization and state, and influences cellular metabolic reprogramming in pluripotent stem cells and non-small cell lung cancer, and can even induce tumorigenesis (Dong et al., 2022; Li et al., 2023). Subsequent investigations have shown that K_*lac*_ modification extends to non-histone proteins in various subcellular compartments and proteins in prokaryotic cells. To date, K_*lac*_ modification has been extensively identified in various organisms, including humans (Cheng et al., 2024; Hong et al., 2023; Lin et al., 2023; Sung et al., 2023; Wu, 2023; Yang Y. H. et al., 2023; Yang Z. et al., 2023; Yao et al., 2023; Zhang et al., 2019), mice (Hagihara et al., 2021; Yang et al., 2022; Huang et al., 2023), rats (Yao et al., 2023), insects (An et al., 2022), plants [rice (Meng et al., 2021) and wheat (Zhu et al., 2023)], fungi [*Botrytis cinerea* (Gao et al., 2020)] and *Phialophora verrucosa* [ Song et al., 2022)], algae [*Nannochloropsis oceanica* (Wang et al., 2023)], parasites [*Toxoplasma gondii* (Yin et al., 2022) and *Trypanosoma brucei* (Zhang et al., 2021)], and bacteria [*Escherichia coli* (Dong et al., 2022) and *Streptococcus mutans* (Li et al., 2023)]. These studies collectively demonstrate the evolutionary conservation of K_*lac*_ modification across diverse organisms. As a reversible PTM, it dynamically regulates protein function, structure, and intracellular interactions, playing crucial roles in various cellular processes such as cellular metabolism, signaling, and gene expression regulation. Moreover, K_*lac*_ modification is associated with pathological conditions. Currently, the investigation of protein K_*lac*_ modification represents an active and burgeoning research area.

Accordingly, *P. damselae* was employed as a representative bacterial strain of the *Vibrioideae* family, and for the first time, we conducted a systematic identification and analysis of K_*lac*_ modification in this bacterium using nano-liquid chromatography-tandem mass spectrometry (nano LC-MS/MS) coupled with affinity purification. A total of 1,352 K_*lac*_ modification sites on 486 proteins were identified, covering various subcellular localizations and diverse biological processes. These findings enhance our understanding of the functional roles of K_*lac*_ modification and provide a dataset for further exploration of K_*lac*_ modification in the physiology and biology of *P. damselae*.

## Materials and methods

### Bacterial strains and growth conditions

*P. damselae* subs. *damselae* was employed in this study. The strain, stored at −80°C, underwent thawing at room temperature. Subsequently, it was activated by inoculating onto Tryptic Soy Broth (TSB) solid agar plates and incubated at 28°C for 24 h. Single colonies obtained were subcultured twice, and PCR analysis was performed on the 16S rDNA and *ureC* genes to confirm the absence of contamination during activation and subculturing. Once the strain was correctly identified, it was inoculated into 200 mL liquid TSB medium and subjected to shaking at 28°C until the OD_600_ of the bacterial culture reached approximately 1.0. The bacterial cells were then collected by centrifugation at 8000 rpm for 5 min at 4°C. The collected cells were washed three times with pre-cooled sterile PBS (137 mM NaCl, 2.7 mM KCl, 10 mM Na_2_HPO_4_, 2 mM KH_2_PO_4_, pH 7.4) at 4°C. Three independently cultured bacterial samples were used as biological replicates.

### Total protein extraction and in-solution trypsin digestion

The preparation of proteins and in-solution trypsin digestion was conducted following the method described in a previous study (Zhu et al., 2021). Briefly, the bacterial pellets obtained were resuspended in a lysis buffer [8 M urea, 2 mM EDTA, 5 mM DTT, and 1% (v/v) protease inhibitor cocktail III (Calbiochem, United States)], and sonicated on ice water. The protein concentration in the supernatant was then quantified using the 2-D Quant kit (GE Healthcare, United States). Subsequently, the protein sample (3 mg) was reduced with 5 mM dithiothreitol (DTT) at 56°C for 30 min, followed by alkylation with 30 mM iodoacetamide (IAM) in the dark at room temperature for 45 min. Afterward, 5 volumes of pre-cooled methanol at −20°C were added to precipitate the proteins, and the mixture was kept at −20°C overnight. The resulting protein precipitate was subsequently centrifuged at 4°C for 10 min, and the protein pellet obtained was washed twice with pre-cooled methanol and placed at −20°C for 1 h. Finally, the proteins were suspended in 0.1 M triethylammonium bicarbonate (TEAB) buffer and digested with trypsin (Promega, United States) at a 1:50 enzyme/substrate ratio for 15 h at 37°C. The tryptic digestion peptides were desalted using a Strata X C18 SPE column (Phenomenex, United States) and then vacuum-dried.

### Affinity enrichment of lysine lactylated peptides

Peptides lactylated at lysine residues were enriched through an immunoprecipitation procedure. Briefly, all of the dried peptides obtained above were redissolved in NETN buffer (100 mM NaCl, 1 mM EDTA, 50 mM Tris-HCl, pH 8.0, and 0.5% NP-40). Subsequently, they were gently rotated overnight at 4°C with anti-lactyl-lysine agarose beads (Micrometer Biotech) at a ratio of 20 μL of beads per milligram of protein. Following enrichment, the supernatant was removed, and the agarose beads were carefully washed three times with NETN buffer and once with double-distilled water. The bound peptides modified with Klac were eluted from the agarose beads with 1% trifluoroacetic acid (TFA), desalted using C18 ZipTips (Millipore, United States), and then vacuum-dried.

### Nano HPLC-MS/MS Analysis

The dried Klac-modified peptides were dissolved in buffer A (2% (v/v) acetonitrile (ACN) and 0.1% (v/v) formic acid (FA) in water), followed by nano-LC-MS/MS analysis as previously described.

The dried Klac-modified peptides (see section 2.2 for modification details) were dissolved in buffer A (2% acetonitrile (ACN), 0.1% formic acid (FA), pH 2.5) and transferred entirely to an autosampler vial for nano LC-MS/MS analysis, performed as previously described.

The resulting dried K_*lac*_-modified peptides were dissolved in buffer A (2% acetonitrile (ACN) and 0.1% formic acid (FA) in water) and then all samples were used for nano LC-MS/MS analysis following a previously described method (Ye et al., 2021) with slight modifications. Initially, the samples were separated by liquid chromatography (LC) using a reversed-phase C18 analytical column (Thermo Acclaim PepMap RSLC C18 column, 75 μm × 500 mm, 2 μm particles) at a flow rate of 250 nL/min on an UltiMate RSLCnano 3000 system (Thermo Scientific, United States). The gradient was set as follows: 2–10% buffer B (0.1% FA and 80% ACN in water) for 6 min, 10–20% buffer B for 45 min, 20–80% buffer B for 7 min, and held at 80% for 4 min. Subsequently, eluted peptides were ionized and electrosprayed (2.0 kV voltage) into the mass spectrometer (Thermo Scientific Q Exactive HFX, United States) coupled online to the LC system. The mass spectrometric analysis was carried out using a data-dependent mode (DDA) on an Orbitrap-based mass analyzer, with automated switching between full MS scans (350–1,800 m/z) and subsequent MS/MS scans. MS spectra were acquired at a resolution of 60,000 (at m/z 200) with an automatic gain control (AGC) target of 1 × 10^6^. The top 15 most intense precursor ions (charge states ≥ 2 +) were selected for fragmentation by higher-energy collision dissociation (HCD) at 26% normalized collision energy (NCE). MS/MS spectra were acquired at a resolution of 30,000 (at m/z 200) with an AGC target of 1 × 10^5^. Dynamic exclusion was implemented for 30 s to minimize repeated sequencing of abundant precursors. All LC-MS/MS experiments were conducted by Micrometer Biotech Co., Ltd. (Hangzhou, China).

### Database searching

All raw data files obtained from mass spectrometry analysis were analyzed using MaxQuant software (v.1.5.2.8). Mass spectra were searched against the protein database of *P. damselae* (29,932 entries) in UniProt. Carbamidomethylation on cysteine was set as a fixed modification, and oxidation on methionine, lactylation on lysine, and lactylation on protein N-terminal were set as variable modifications. The false discovery rate (FDR) thresholds for peptides, proteins, and modification sites were set at < 1%. Trypsin was set as a cleavage enzyme with up to 4 missing cleavages, and the minimum number of amino acids for peptides was set at 7. The site localization probability for Klac sites was set at > 0.75, and the minimum score for modified peptides was set at > 40. Mass tolerances for precursor ions and fragment ions were set at 10 ppm and 0.02 Da, respectively.

### Bioinformatics analysis of lysine lactylated peptides and proteins

To investigate the potential functions of identified Klac-modified peptides and proteins, comprehensive bioinformatics analyses were carried out. Functional classification and enrichment analysis of Gene Ontology (GO) were conducted using the UniProt-GOA database^1^ (Dimmer et al., 2012). For functional annotation of the Kyoto Encyclopedia of Genes and Genomes (KEGG), the KEGG Automatic Annotation Server (KAAS) tool (v.2.0) was used for pathway annotation, and the online service tool KEGG Mapper was used for mapping (Kanehisa and Goto, 2000). GO term or KEGG pathway enrichment analysis was performed using the DAVID tool (Dennis et al., 2003), and annotation terms with a corrected *p*-value < 0.05 by Fisher’s exact test were considered significantly enriched. Subcellular location analysis of identified proteins was performed using the WoLF PSORT platform.^2^ Potential protein-protein interaction relationships among identified proteins were assessed using STRING (v.12),^3^ and only relationships with a confidence score ≥ 0.7 were considered significant. The protein-protein interaction (PPI) network was constructed and visualized using Cytoscape software (v.3.8) (Shannon et al., 2003), and modules of the PPI network were identified using the Molecular Complex Detection (MCODE) plug-in tool in Cytoscape.

## Results and discussion

### Global identification of K_*lac*_ modification in *P. damselae*

Although K_*lac*_ modification of proteins was firstly discovered on histones in mammalian cells (Zhang et al., 2019), in recent years, this modification has also been successively found to be present in a wide variety of other organisms, including insects, plants, fungi and bacteria. In bacteria, global identification and characterization of K_*lac*_-modified proteins have only been carried out in *E. coli* (Dong et al., 2022) and *S. mutans* (Li et al., 2023). A total of 1,047 K_*lac*_ sites on 478 proteins were identified in the lactylome of *E. coli*, while in the lactylome of *S. mutans*, a total of 1,869 K_*lac*_ sites on 469 proteins were identified under both low- and high-sugar conditions.

**FIGURE 1:**
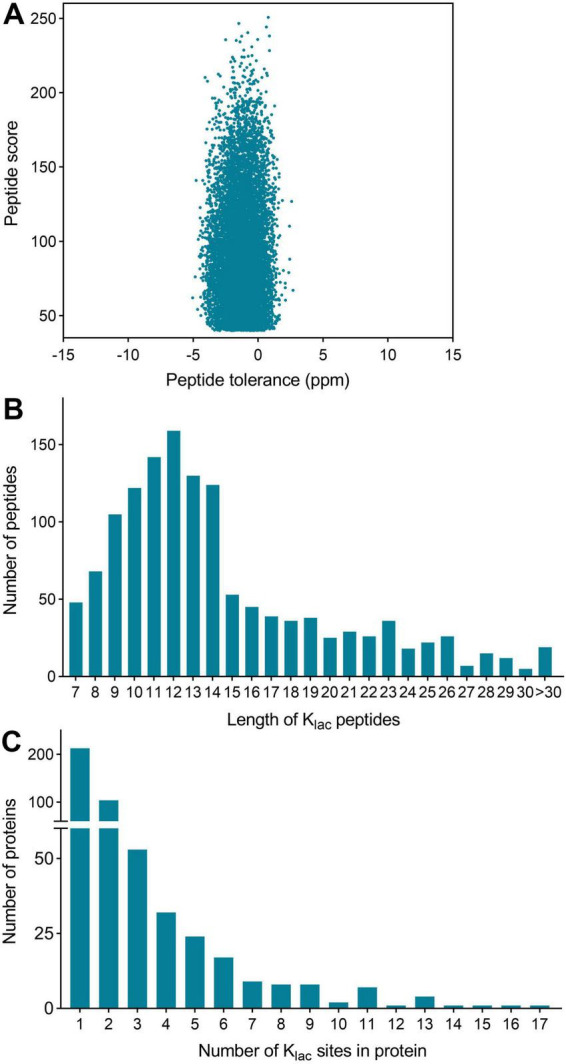
Global analysis of K_*lac*_-modified peptides and proteins identified in *P. damselae*. **(A)** Peptide tolerance (−5 to 2 ppm) and peptide score (40–264) distribution of all identified K_*lac*_-modified peptides. **(B)** Peptide length distribution of all K_*lac*_-modified peptides. **(C)** Protein distribution of K_*lac*_-modified sites contained in each protein.

*P. damselae* is a marine bacterium belonging to the Vibrionaceae family, which has been implicated in a variety of fish diseases across its species. Additionally, bacteria in this family possess tremendous metabolic and genetic diversity (Soto, 2022). Therefore, conducting studies on PTM of proteins, including K_*lac*_ modification, in bacteria of this family contributes to understanding the mechanisms underlying their diversity. In the present study, K_*lac*_ modifications in *P. damselae* were identified and systematically analyzed. Total proteins from *P. damselae* were extracted, and a proteomic research strategy involving affinity enrichment using K_*lac*_ pan-antibodies and nano LC-MS/MS was employed for K_*lac*_-modified protein identification, with three biological replicates performed for verification. After conducting three repeated tests, we identified a total of 1,352 K_*lac*_ modification sites on 486 proteins. These sites had a localization probability greater than 0.75 in at least one sample across the three replicates (Supplementary Table S1). The identified K_*lac*_-modified peptides had scores exceeding 40, and the mass error was within 5 ppm (Figure 1A), meeting the high precision requirements of orbitrap mass spectrometry. This indicates that the mass accuracy of the mass spectrometer is normal and does not adversely affect the qualitative or quantitative analysis of proteins due to excessive mass deviation. Therefore, the identification results meet the requirements for further analysis.

**FIGURE 2:**
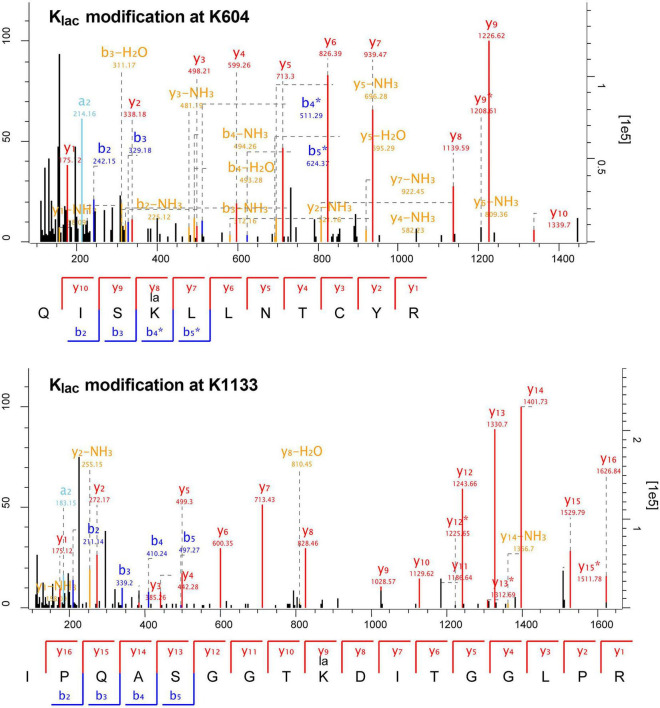
Representative MS/MS spectra of K_*lac*_-modified peptides from DNA-directed RNA polymerase subunit β’ (UniProt accession: KFZ67_13710). The fragmentation patterns of the K_*lac*_-modified peptides QISKlacLLNTCYR (K604) and IPQASGGTKlacDITGGLPR (K1133) are displayed below their corresponding spectra. Characteristic b and y ions are labeled, with b ions in blue and y ions in red.

The length distribution of the K_*lac*_-modified peptides was analyzed and found to vary within the range of 7 and 42 amino acid residues, in accordance with the general pattern based on trypsin enzymatic digestion and HCD fragmentation. The majority of these peptides fell within the range of 8–15 residues, constituting 67% of the total identified K_*lac*_-modified peptides. Each length category had more than 50 peptides (Figure 1B and Supplementary Table S1). Furthermore, the identified K_*lac*_-modified peptides were matched to a total of 486 proteins. The range of K_*lac*_ modification sites in these proteins varied from 1 to 17, where 44% of proteins had 1 K_*lac*_ modification site, and proteins with 2–5 K_*lac*_ modification sites accounted for 21, 11, 7, and 5% respectively. Proteins with more than 5 K_*lac*_ modification sites constituted 12%, with only 3% having more than 10 sites (Figure 1C and Supplementary Table S1). Notable examples include 50S ribosomal protein L13 with 14 sites (UniProt accession: KFZ67_01775), chaperone protein DnaK with 15 sites (KFZ67_13505), 60 kDa chaperonin with 16 sites (KFZ67_11605), and DNA-directed RNA polymerase subunit β’ with 17 sites (KFZ67_13710) (Supplementary Table S1). The representative MS/MS spectra of K_*lac*_-modified peptides at positions K604 and K1133 of the DNA-directed RNA polymerase subunit β’ are shown in Figure 2.

The number of K_*lac*_-modified proteins we identified in *P. damselae* is comparable to that in two other bacteria, *E. coli* (478) (Dong et al., 2022) and *S. mutans* (469) (Li et al., 2023). However, there is a significant difference in the number of K_*lac*_ modification sites, with 1,352, 1,047, and 1,869 in *P. damselae*, *E. coli*, and *S. mutans*, respectively. This indicates a distinct distribution of K_*lac*_ modification sites on individual proteins among these three strains. The variation in K_*lac*_ modification site numbers may reflect inherent differences in the lactylation levels of proteins among different bacterial species. However, it cannot be excluded that such differences are result from separate identifications conducted by different laboratories, despite the adoption of similar proteomic identification strategies.

The identification of so many K_*lac*_-modified sites and proteins in these bacteria not only indicates that K_*lac*_ modification is also an abundant and complex PTM in prokaryotic cells but also underscores its crucial role in regulating protein functions in these bacteria. Particularly noteworthy is the widespread K_*lac*_ modification observed in ribosomal proteins and RNA polymerase. Additionally, Pan et al. (2015) previously identified extensive succinylation modification on these two types of proteins in *V. parahaemolyticus*, suggesting a stringent control of protein translation in bacteria. In a word, this study represents the first investigation into K_*lac*_ modification in *P. damselae*, and the obtained K_*lac*_-modified proteomic data will contribute to further elucidating the regulatory role of K_*lac*_ modification in the physiological functions of this bacterium.

### Gene Ontology (GO) functional annotation and subcellular localization distribution of the K_*lac*_-modified proteins in *P. damselae*

To gain a comprehensive understanding of the K_*lac*_-modified proteins identified in *P. damselae*, we conducted a thorough analysis of these proteins in terms of gene ontology (GO) annotation and subcellular localization. This is important because K_*lac*_-modified protein plays a vital role in regulating cellular functions (Pan et al., 2015). GO serves as a crucial bioinformatics analysis method and tool, employed to describe various attributes of genes and gene products (Dimmer et al., 2012). GO annotations comprise three dimensions: biological process, cellular component, and molecular function, offering diverse perspectives on the biological roles of proteins.

**FIGURE 3:**
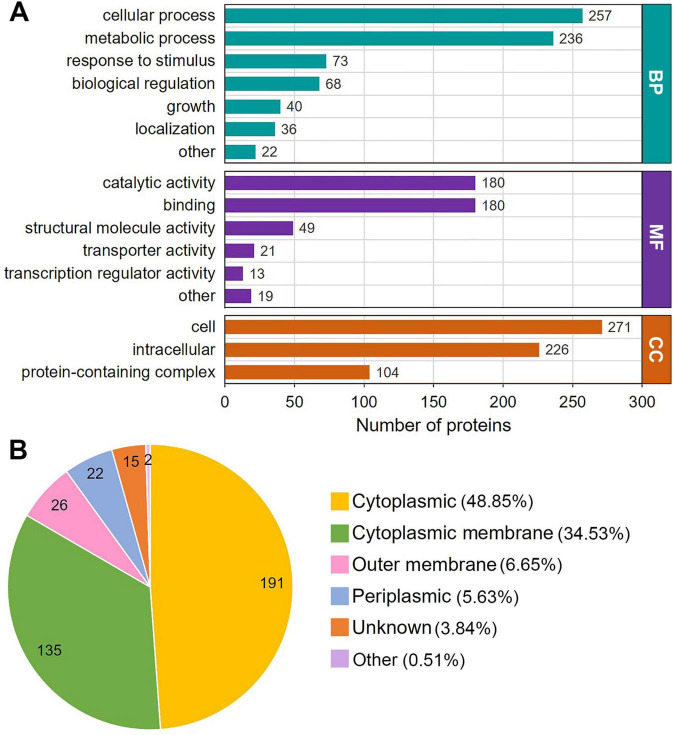
Gene Ontology (GO) functional annotation and subcellular localization distribution of the K_*lac*_-modified proteins in *P. damselae*. **(A)** GO annotation based on biological process (BP), cellular component (CC) and molecular function (MF). The number of proteins in each category is indicated. **(B)** Subcellular localization distribution of K_*lac*–_modified proteins.

The results of the GO functional classification analysis are shown in Figure 2A and Supplementary Table S2. In the classification of biological processes, K_*lac*_-modified proteins are mainly associated with cellular processes and metabolic processes, with protein numbers of 257 and 236, respectively, significantly exceeding those ranked 3-5 in response to stimulus, biological regulation, growth and localization with 73, 68, 40 and 36 proteins, respectively (Figure 3A, green bars). Classification by molecular function revealed that the majority of K_*lac*_-modified proteins are associated with catalytic activity and binding, with 180 proteins for both categories, which was also significantly higher than the proteins with other functions (Figure 3A, purple bars), consistent with the classification of biological processes. These results suggest that K_*lac*_ modification may play a critical role in cellular processes and metabolic processes. In the cellular component classification, K_*lac*_-modified proteins are mainly categorized into cell and intracellular (Figure 3A, orange bars).

Moreover, the subcellular localization of all identified K_*lac*_-modified proteins was analyzed using the WoLF PSORT software, which showed that about half of the proteins (48.85%) are located in the cytoplasm, while the percentage of proteins located in the cytoplasmic membrane is also high at 34.53%, and in contrast, proteins in the outer membrane and periplasmic space constitute only a small fraction, accounting for 6.65 and 5.63%, respectively (Figure 3B and Supplementary Table S3). Taken together, these findings suggest that K_*lac*_-modified proteins are widely distributed throughout the cell, and are involved in a diverse variety of functions and consequently participating in various biological processes.

### Enrichment analysis of K_*lac*_-modified proteins in *P. damselae*

**FIGURE 4:**
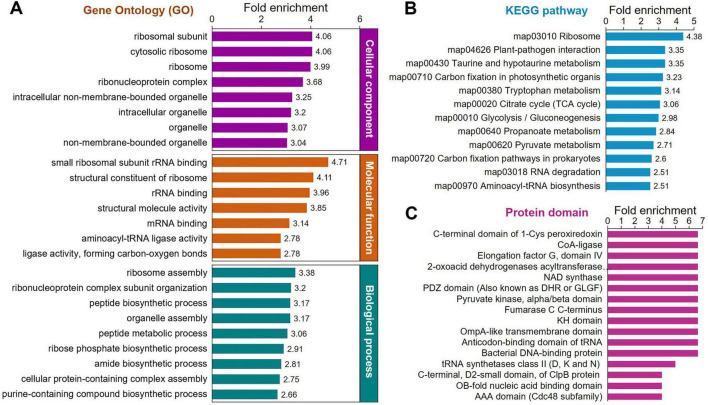
Protein enrichment analysis of the K_*lac*_-modified proteins in *P. damselae*. **(A)** GO enrichment for biological process (*P* < 10^–3^), molecular function (*P* < 10^–7^) and cellular components (*P* < 10^–11^). **(B)** KEGG pathway enrichment (*P* < 10^–3^). **(C)** Enrichment for protein domains (*P* < 10^–3^).

To better understand the functions of K_*lac*_-modified proteins in *P. damselae* and to investigate whether K_*lac*_ modification shows significant enrichment in specific functional categories, we performed further enrichment analysis on the identified K_*lac*_-modified proteins in terms of GO annotation, KEGG pathways, and protein domains. The results demonstrate a significant enrichment of K_*lac*_-modified proteins in these categories (Figure 4 and Supplementary Tables S4–S6). The molecular function-based GO enrichment analysis reveals significant enrichment in functions such as small ribosomal subunit, rRNA binding, structural constituent of ribosome, structural molecule activity, mRNA binding, and aminoacyl-tRNA ligase activity (Figure 4A, orange bars, and Supplementary Figure S1). Interestingly, these significantly enriched molecular functions, with the exception of structural molecule activity and ligase activity (forming carbon-oxygen bonds), are all related to ribosome and protein biosynthesis. The GO enrichment analysis of cellular components reveals that the top 4 highly enriched cellular components, including ribosomal subunit, cytosolic ribosome, ribosome, and ribonucleoprotein complex, are also closely associated with ribosome and protein biosynthesis. However, other cellular components, such as intracellular non-membrane-bounded organelle, intracellular organelle, organelle, and non-membrane-bounded organelle, also exhibit a certain degree of enrichment (Figure 3A, purple bars, and Supplementary Figure S1). Consistent with these observation, in the biological process-based GO enrichment analysis, K_*lac*_-modified proteins are significantly enriched in processes related to ribosome and protein synthesis, including the top 3 highly enriched processes: ribosome assembly, ribonucleoprotein complex subunit organization, and peptide biosynthetic process (Figure 4A, green bars, and Supplementary Figure S1). The remaining enriched processes mainly involve nucleic acid metabolism (such as ribose phosphate biosynthetic process, assembly purine-containing compound biosynthetic process, ribose phosphate metabolic process, and nucleoside phosphate metabolic process) and protein metabolism (including peptide metabolic process, cellular protein-containing complex assembly, and cellular protein metabolic process, etc.). These GO enrichment analysis results suggest that proteins associated with ribosome, protein biosynthesis, and metabolism are more prone to undergo K_*lac*_ modification in *P. damselae*, indicating that K_*lac*_ modification plays a more crucial regulatory role in these physiological functions compared to other functions.

**FIGURE 5:**
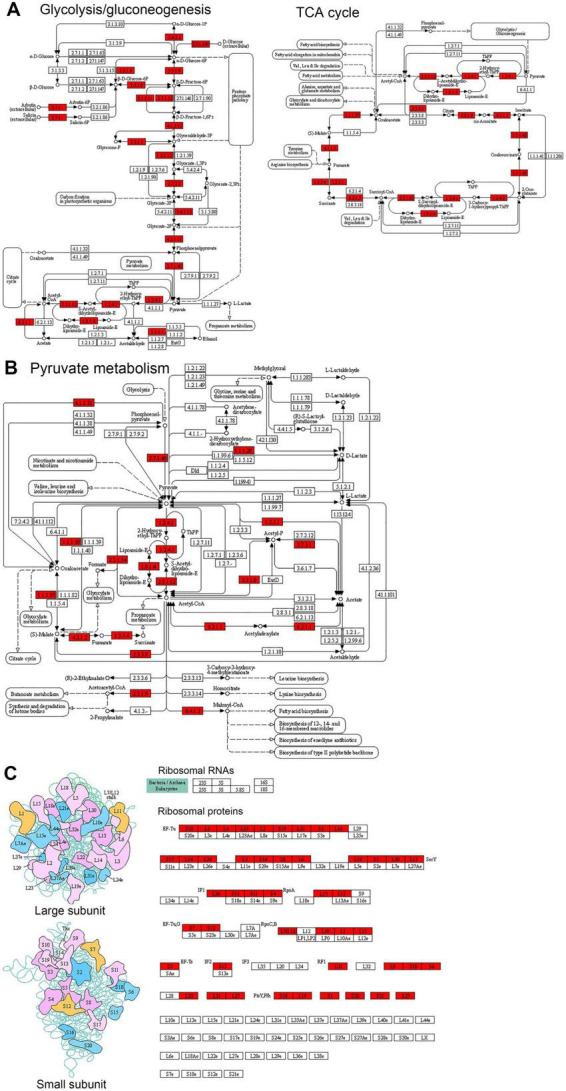
Representative KEGG pathways showing significant enrichment of K_*lac*_-modified proteins in *P. damselae*. **(A)** Central metabolism, including glycolysis/gluconeogenesis, and TCA cycle and oxidative phosphorylation. **(B)** Pyruvate metabolism. **(C)** Ribosome. The K_*lac*_-modified enzymes are indicated by the red background.

Enrichment analysis of KEGG pathways indicates significant enrichment in several pathways related to ribosome, protein biosynthesis, and metabolism (Figure 4B, Supplementary Figure S2, and Supplementary Table S5). Among these enriched pathways, ribosome, and aminoacyl-tRNA biosynthesis are associated with ribosome and protein biosynthesis (K_*lac*_-modified proteins in the ribosome are shown in Figure 5C, and K_*lac*_-modified proteins in the aminoacyl-tRNA biosynthesis pathway are shown in Supplementary Figure S3). Additionally, pathways involved in central carbon metabolism, such as TCA cycle, glycolysis/gluconeogenesis, pentose phosphate pathway, pyruvate metabolism, and glyoxylate and dicarboxylate metabolism, exhibit significant enrichment (K_*lac*_-modified proteins in glycolysis/gluconeogenesis and TCA are shown in Figure 5A, and K_*lac*_-modified proteins in pyruvate metabolism are presented in Figure 5B). Other enriched pathways also mostly involve metabolic processes, such as amino acid metabolism and nucleotide metabolism (Supplementary Figure S2). These findings are largely consistent with the aforementioned GO enrichment results.

Furthermore, we performed enrichment analysis on the protein domains of K_*lac*_-modified proteins. Protein domains refer to certain structural elements recurring in different proteins, often having similar sequence, structure and function, and they serve as the evolutionary units of proteins. As shown in Figure 4C, Supplementary Figure S2, and Supplementary Table S6, the enrichment results further confirm that proteins involved in protein biosynthesis and metabolism are more prone to be K_*lac*_-modified. Therefore, these diverse enrichment analysis results mentioned above collectively suggest that K_*lac*_ modification plays a crucial regulatory role in physiological functions related to protein biosynthesis and metabolism in *P. damselae*.

It is noteworthy that GO enrichment analysis of identified K_*lac*_-modified proteins in *S. mutans* suggests that these proteins are mainly enriched in biological processes related to translation, peptide biosynthesis, and amide biosynthesis (Li et al., 2023). The molecular functions of K_*lac*_-modified proteins are enriched in structural molecular activity, and structural composition of ribosomes. KEGG pathway analysis indicates K_*lac*_-modified proteins closely associated with ribosome, aminoacyl-tRNA synthesis, and glycolysis/gluconeogenesis pathways (Li et al., 2023). In *E. coli*, proteins related to metabolism, translation, ribosome assembly, and biosynthesis are significantly enriched (Dong et al., 2022). The enrichment analysis results for K_*lac*_-modified proteins in these two bacteria are remarkably similar to the results obtained in this study for *P. damselae*. Combined with the available data for K_*lac*_-modified proteins in these three different bacteria, it is reasonable to suggest that K_*lac*_ modification of proteins associated with protein biosynthesis and central carbon metabolism may represent an important regulatory pattern in the control of physiological functions.

### Analysis of protein interaction networks of K_*lac*_-modified proteins in *P. damselae*

**FIGURE 6:**
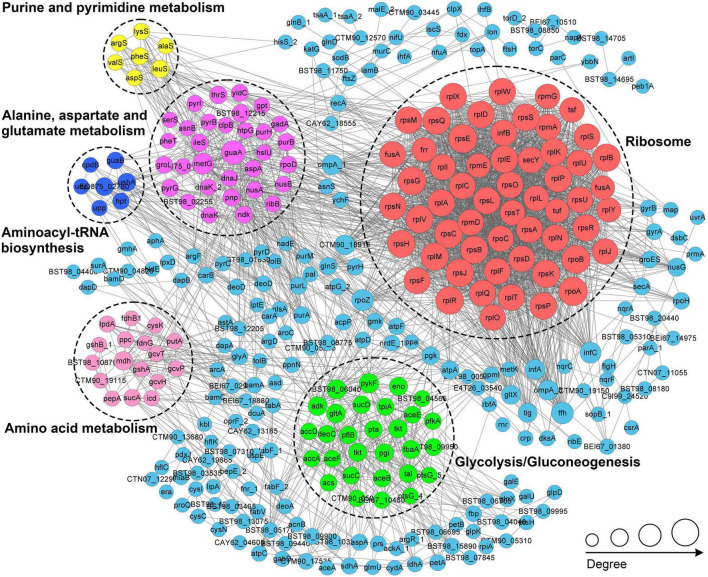
PPI network analysis of the K_*lac*_-modified proteins in *P. damselae*. Representatives of the six PPI clusters enriched in ribosomes (red), glycolysis/glycogenesis (green), alanine, aspartate, and glutamate metabolism (purple), amino acid metabolism (pink), aminoacyl-tRNA biosynthesis (blue), and purine and pyrimidine metabolism (yellow), respectively, are shown in different colors.

We conducted a protein-protein interaction network (PPI) analysis of all identified K_*lac*_-modified proteins using the STRING database and Cytoscape software (Smoot et al., 2011) for a better understanding of the cellular processes regulated by K_*lac*_ modification in *P. damselae*. As a result, 283 K_*lac*_-modified proteins were identified as network nodes, and 4868 interactions with their scores of ≥ 0.90 were obtained from the STRING database (Supplementary Table S7). The global PPI network diagram for these interactions is shown in Figure 6, and detailed interaction information can be found in Supplementary Figure S4 and Supplementary Table S7. Furthermore, 20 PPI clusters were extracted from the global PPI network by a clustering algorithm executed using the MCODE plug-in toolkit in Cytoscape (Supplementary Table S7), including highly connected clusters related to ribosomes, glycolysis/gluconeogenesis, purine and pyrimidine metabolism, aminoacyl-tRNA biosynthesis, and amino acid metabolism, each represented by a different color in Figure 6. These results suggest that K_lac_ modification tends to occur on proteins associated with specific functional clusters. Notably, the extracted clusters are consistent with GO annotations and KEGG pathway enrichment analysis.

In *S. mutans*, the PPI network of K_*lac*_-modified proteins was also analyzed, where the top 5 clusters were mainly enriched for ribosomes, glycolysis/gluconeogenesis, PTS, sulfur metabolism, and aminoacyl-tRNA synthesis (Li et al., 2023). Among these clusters, those involving ribosomes, glycolysis/gluconeogenesis, and aminoacyl-tRNA synthesis were also found to be enriched in the present study. In *E. coli*, despite the identification of the lactylome, the K_*lac*_-modified proteins were not subjected to PPI network analysis. However, given the high homology (68.9%) between CobB, the enzyme identified as the primary regulator of K_lac_ modification in *E. coli* (Li et al., 2023), and CobB in *P. damselae* (Supplementary Figure S5), it is highly probable that PPI network analysis would result in predominantly enriched clusters related to ribosome/protein biosynthesis and central metabolism. Since PTMs with dynamic and reversible properties can impact the PPI network (Rao et al., 2014), the results of these PPI analyses suggest that K_lac_ modifications may regulate these relevant physiological processes and metabolic pathways by influencing PPIs, thereby modulating cellular physiological functions.

## Conclusion

In this study, we present the first systematic analysis of K_lac_ modification in *P. damselae* using a highly sensitive proteomic approach. A total of 1,352 K_*lac*_-modified sites corresponding to 486 proteins were identified in *P. damselae*, and analysis of the GO annotation, KEGG pathway and PPI network of K_*lac*_-modified proteins indicates their involvement in various cellular functions and metabolic pathways, particularly in ribosome and protein biosynthesis, and central carbon metabolism. This suggests that K_lac_ modification is an abundant and conserved PTM in *P. damselae*, similar to the other two bacteria or eukaryotic organisms that have undergone global K_lac_ identification. Although the potential regulatory roles and mechanisms of K_lac_ modification in *P. damselae* remain to be elucidated, our K_*lac*_-modified protein dataset will help to better reveal the role of K_lac_ modification in regulating the physiological functions of *P. damselae*.

## Data Availability

The datasets presented in this study can be found in online repositories. The names of the repository/repositories and accession number(s) can be found in the article/Supplementary material.

## Footnotes

1
http://www.ebi.ac.uk/GOA/

2
https://wolfpsort.hgc.jp

3
https://cn.string-db.org/
