# Supplementary material for: Global analysis of protein lysine lactylation profiles in the marine bacterium Photobacterium damselae subsp. damselae
Source: Front Microbiol. 2025 Jun 17;16:1539893. doi: 10.3389/fmicb.2025.1539893 (PMC12209183; doi:10.3389/fmicb.2025.1539893)
Supplement: Supplementary file 1 [file Data_Sheet_1.docx]

**Supplementary information**

**Global analysis of protein lysine lactylation profiles in the marine bacterium *Photobacterium damselae***

Yongxiang Yu1^,2^, Haozhe Liu1^,2^, Chunyuan Wang^1,2^, Yingeng Wang^1,2^, Xiaojun Rong ^1,2^, Meijie Liao ^1,2^, Bin Li^1,2^, Xingling Yi^3^, and Zheng Zhang^1,2*^

*^1^ Yellow Sea Fisheries Research Institute, Chinese Academy of Fishery Sciences, State Key Laboratory of Mariculture Biobreeding and Sustainable Goods, Qingdao 266071, Shandong, China*

*^2^ Laboratory for Marine Fisheries Science and Food Production Processes, Laoshan Laboratory, Qingdao 266237, Shandong, China*

*^3^ Weimi Biotechnology Co. Ltd., Hangzhou 310051, Zhejiang, China*

* Corresponding author: Zheng Zhang (E-mail: zhangzheng@ysfri.ac.cn)

**Contents**

1. Supplemental Figures and Figure Legends

2. Supplemental Tables

1. **Supplemental Figures and Figure Legends**


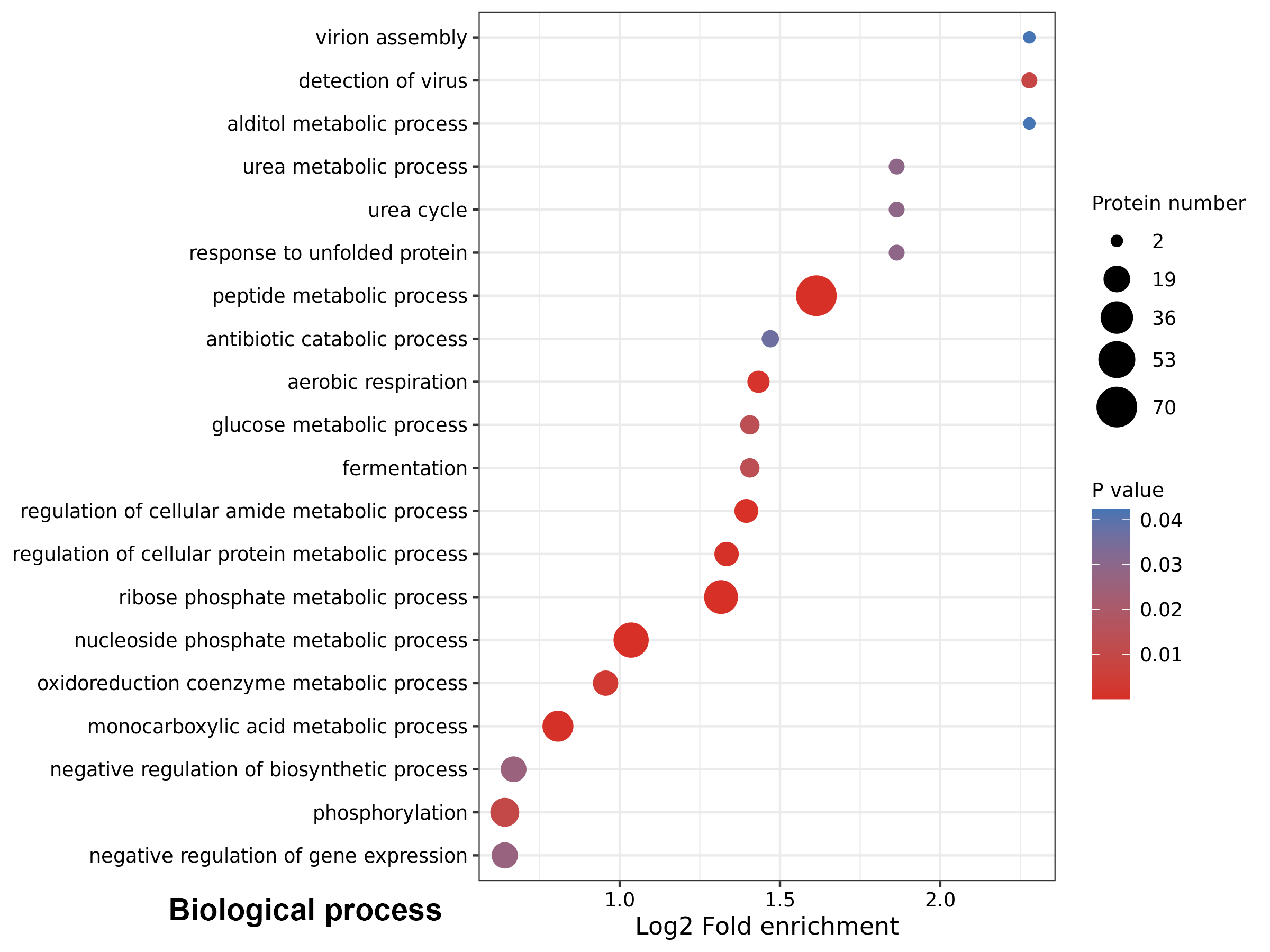


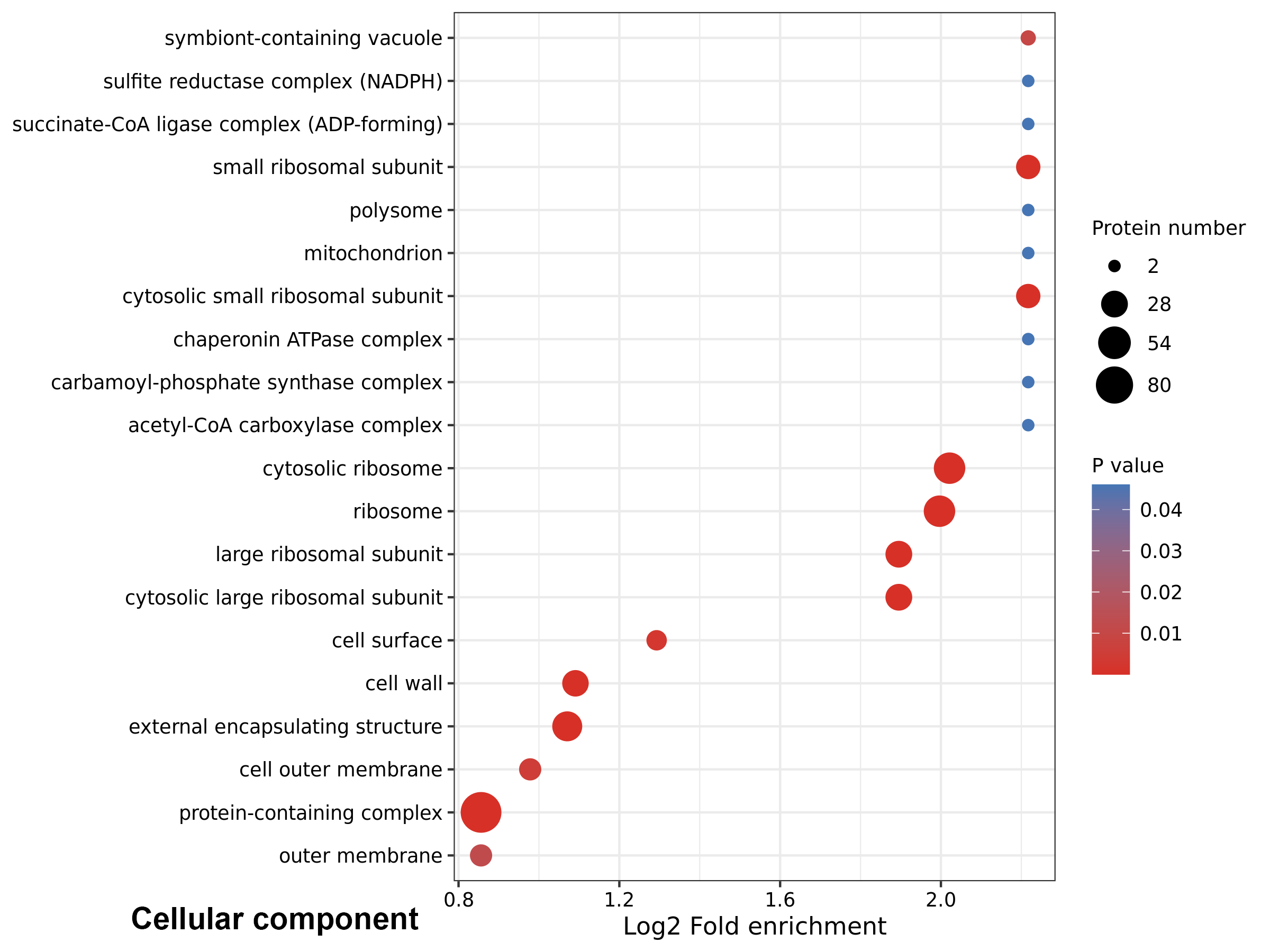


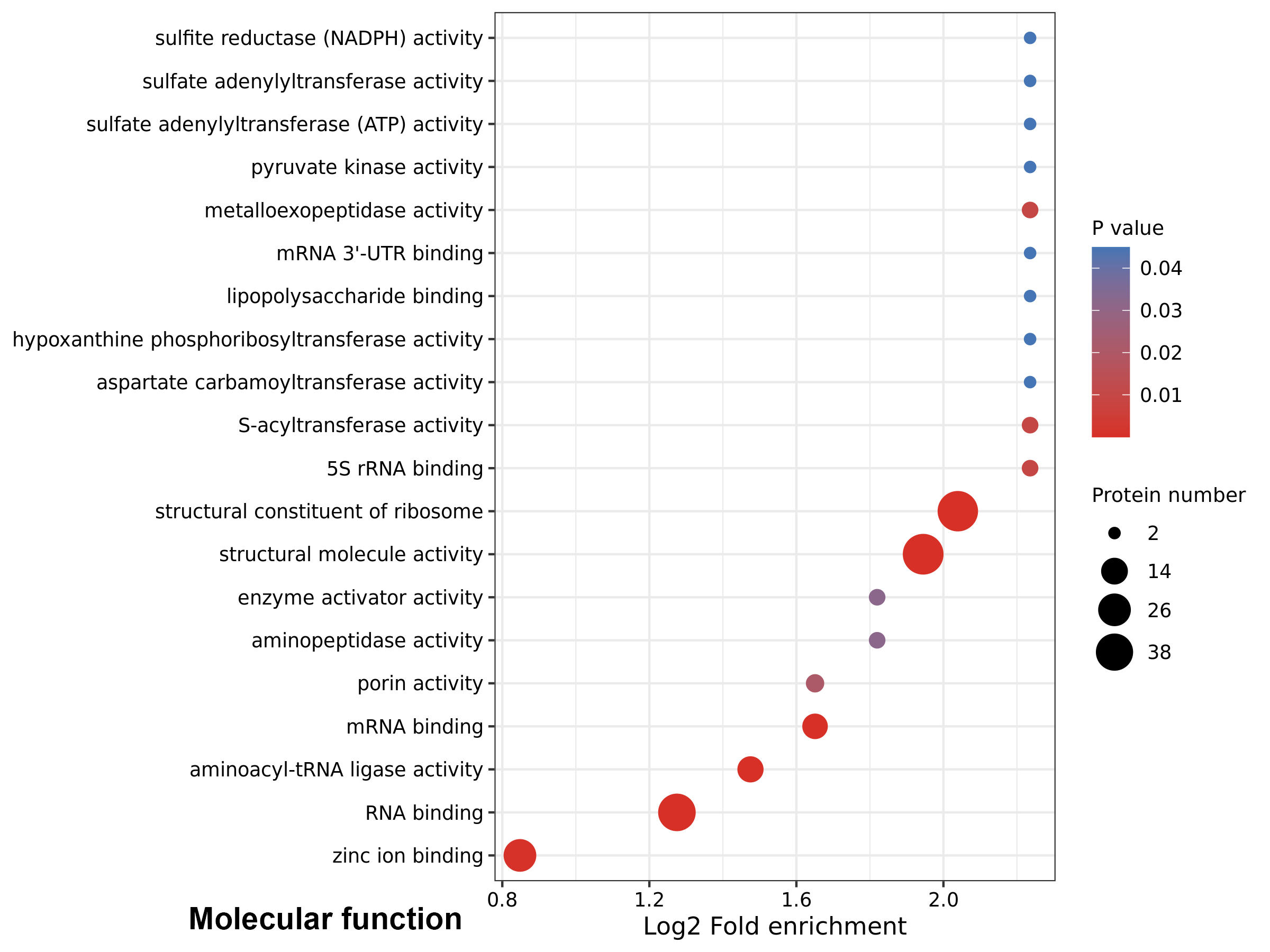


**Figure S1.** **The bubble charts show the GO enrichment analyses for biological process, molecular function and cellular component of K_lac_-modified proteins in *P. damselae*.** The p-values obtained from the enrichment analysis (utilizing Fisher's exact test) for significant enrichment of protein modification (p < 0.05) are visually presented in a bubble chart according to functional categories. The bubble chart shows the top 20 most significantly enriched categories. The vertical axis of the bubble chart represents the functional categories, while the horizontal axis represents the Log2-transformed values of the fold enrichment of the proportion of modified proteins compared to the proportion of identified proteins within each functional category.


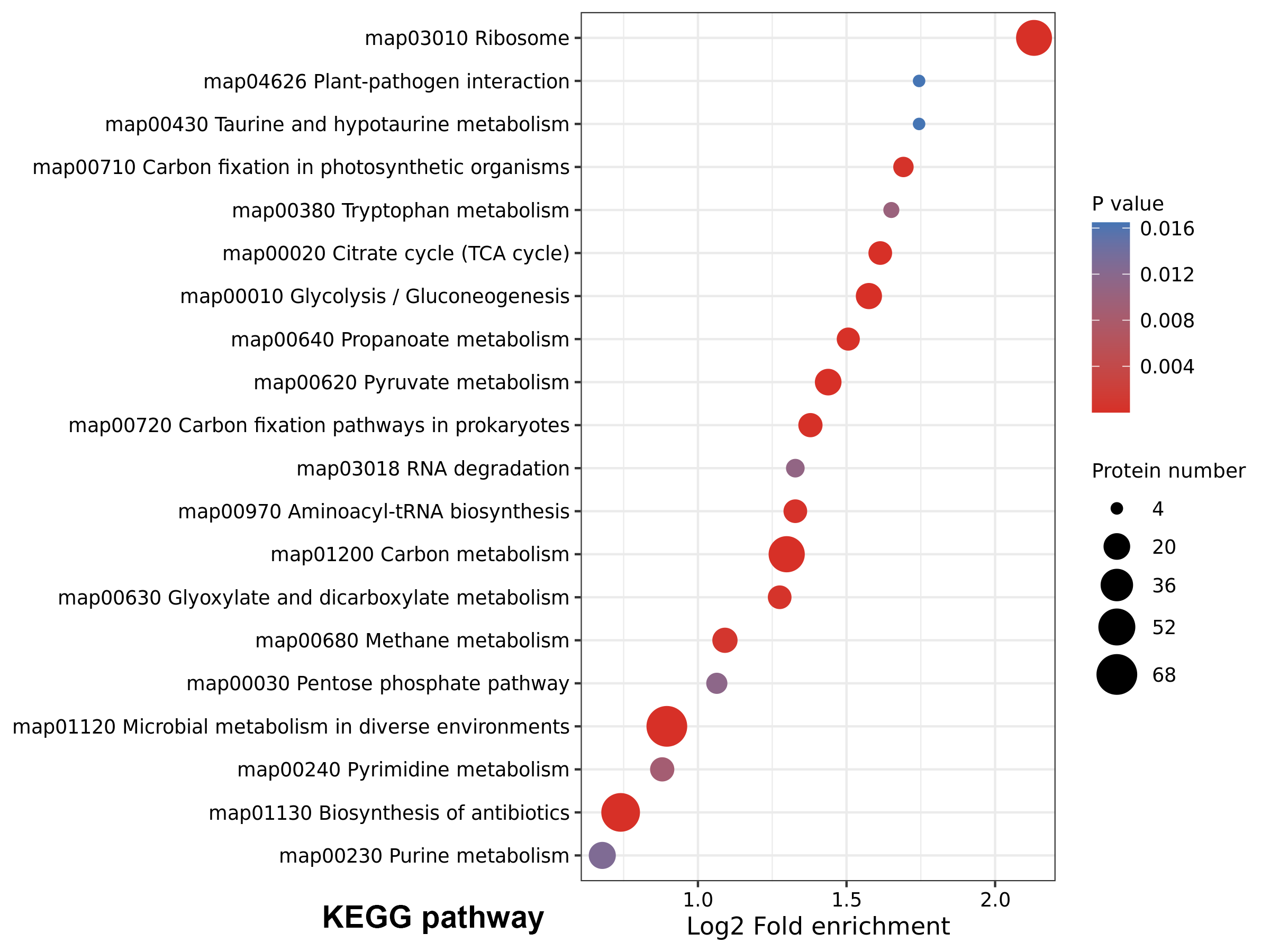


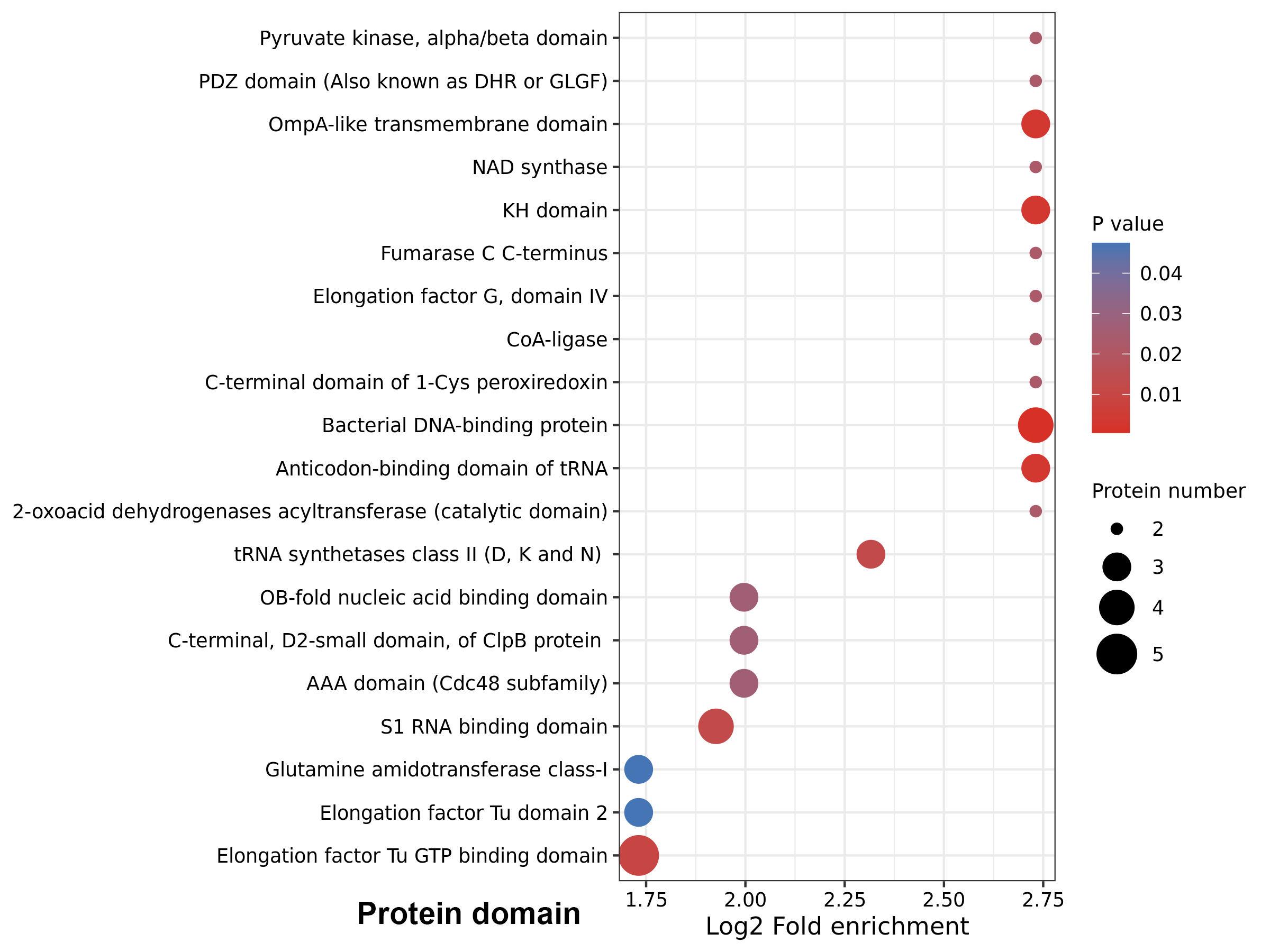


**Figure S2. The bubble charts show the enrichment of KEGG pathway and protein domain of K_lac_-modified proteins in *P. damselae***. The p-values obtained from the enrichment analysis (using Fisher's exact test) for significant enrichment of protein modification (p < 0.05) are visually presented in a bubble chart according to pathways or protein domains. The bubble chart shows the top 20 significantly enriched categories. The vertical axis of the bubble chart represents the functional categories, while the horizontal axis shows the Log2-transformed values of the fold enrichment of the proportion of modified proteins compared to the proportion of identified proteins within each functional category.


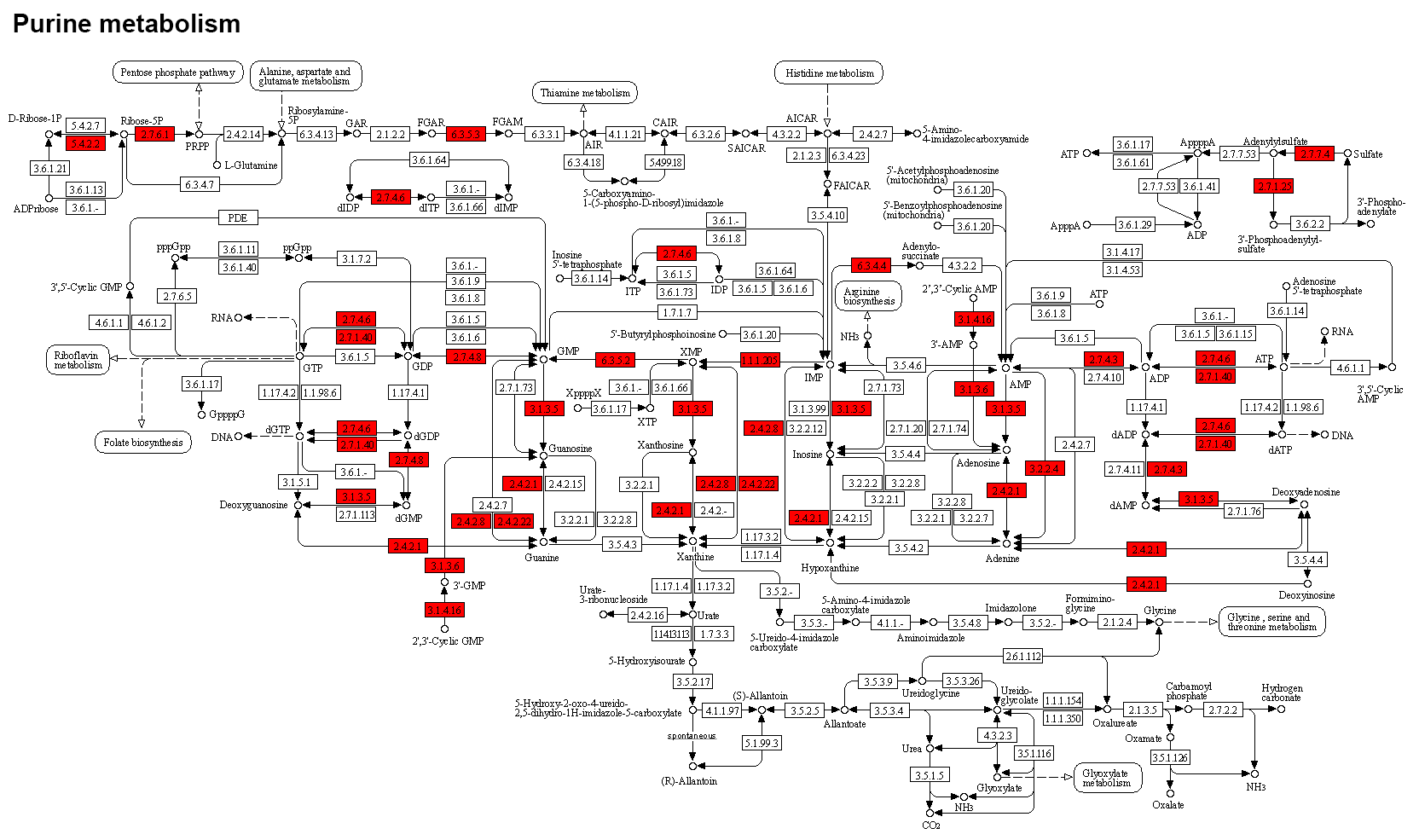

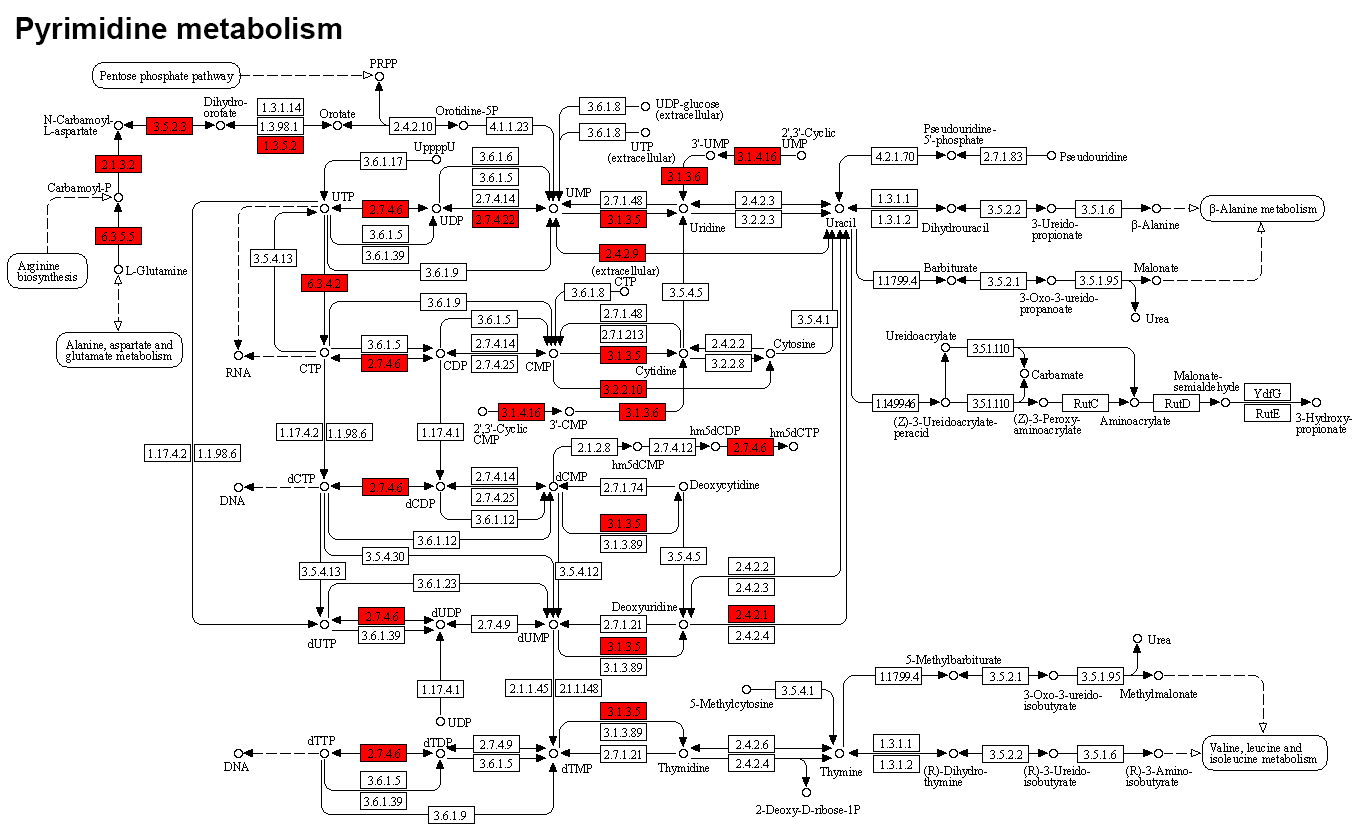

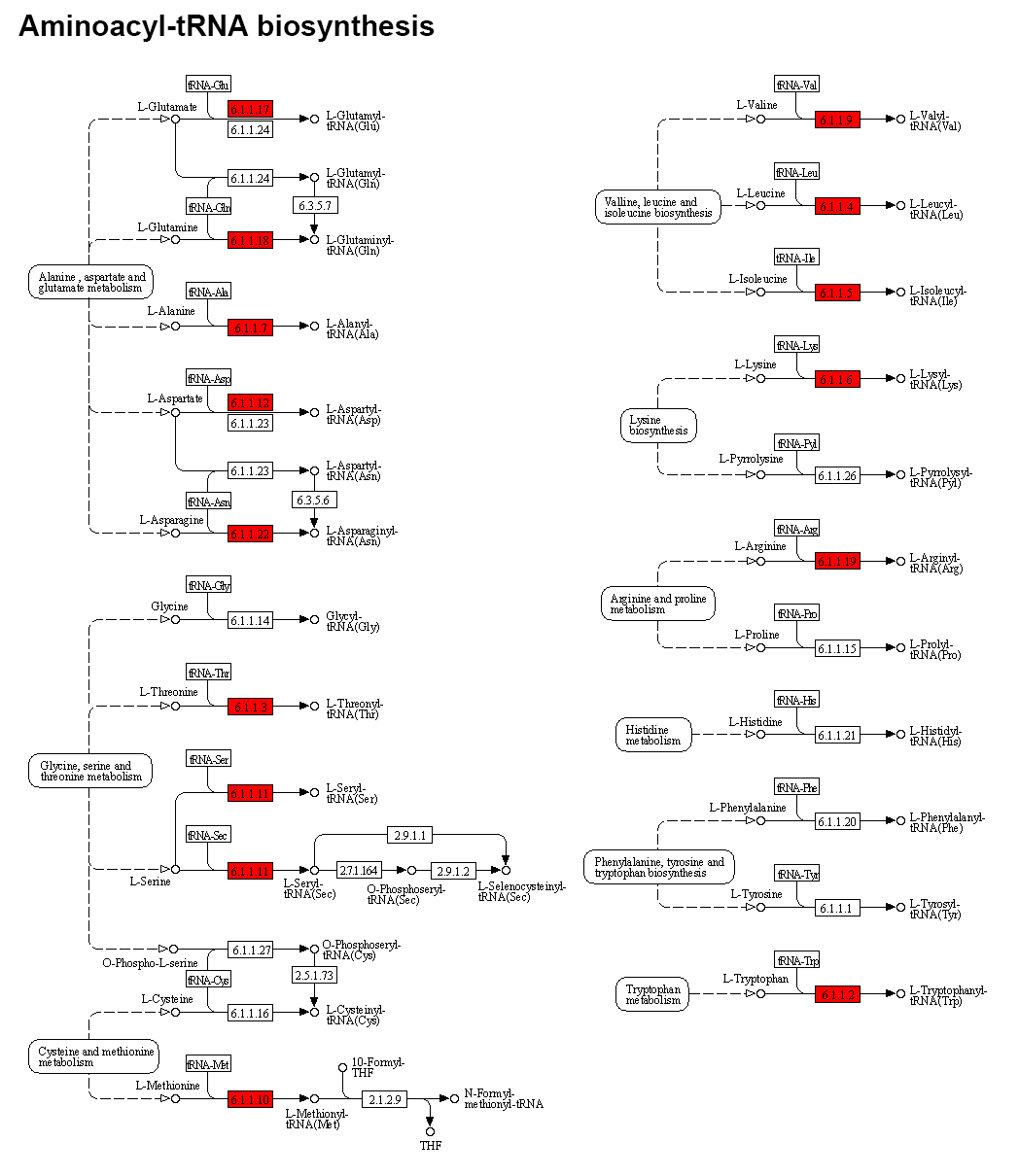

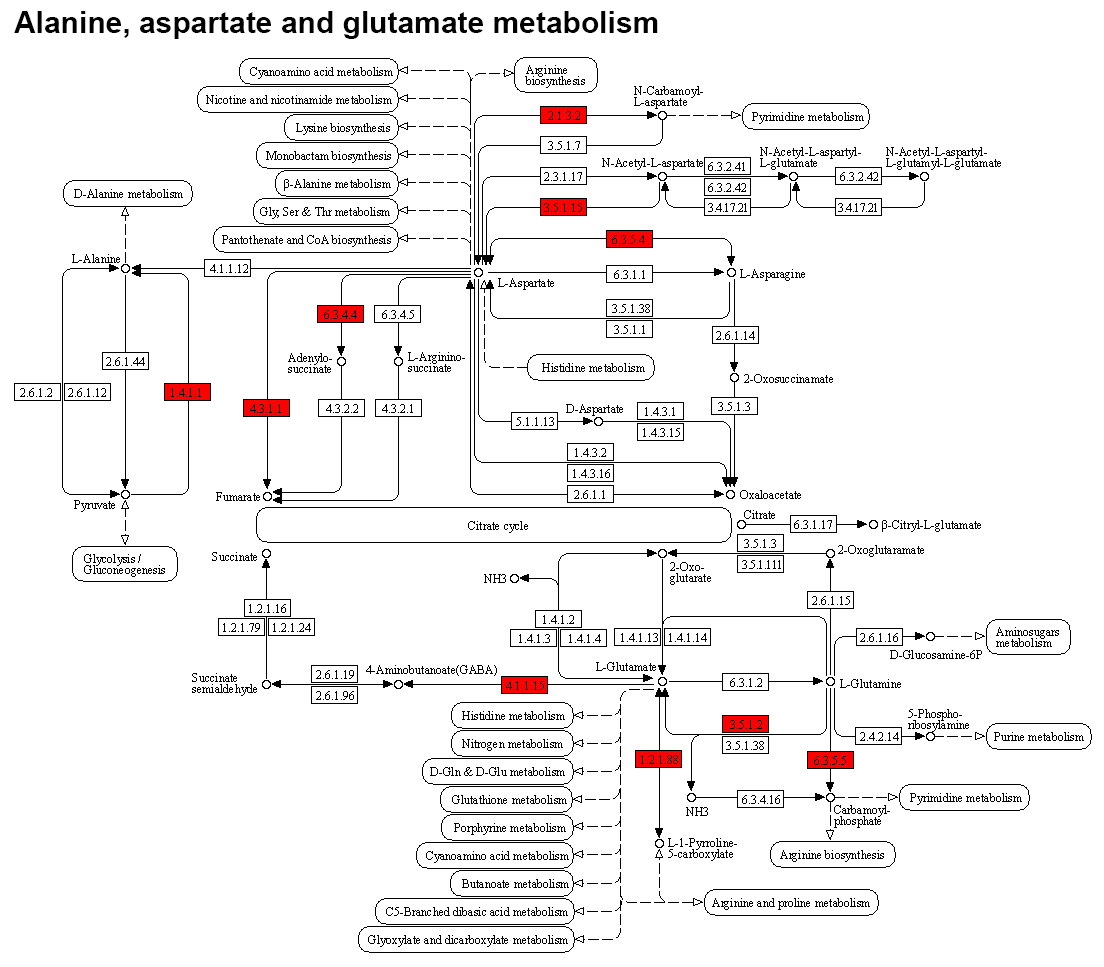


**Figure S3. Representative KEGG pathway maps of K_lac_-modified proteins in *P. damselae***. K_lac_-modified proteins are enriched in several KEGG pathways including purine metabolism, pyrimidine metabolism, aminoacyl-tRNA biosynthesis, and alanine, aspartate, and glutamate metabolism. K_lac_-modified proteins are shown in red boxes.


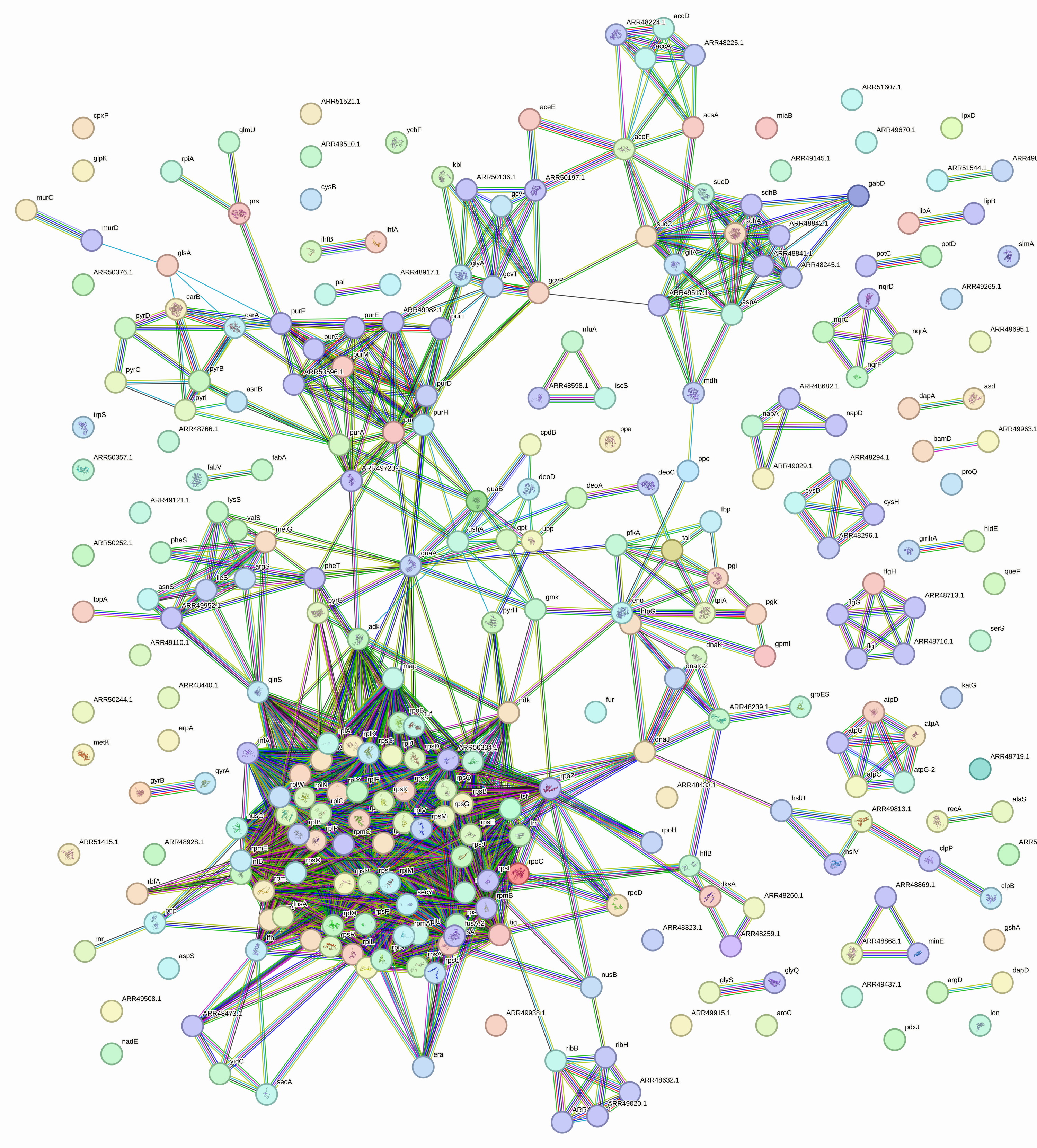


**Figure S4. The global PPI network of K_lac_-modified proteins identified in *P. damselae*.**

**
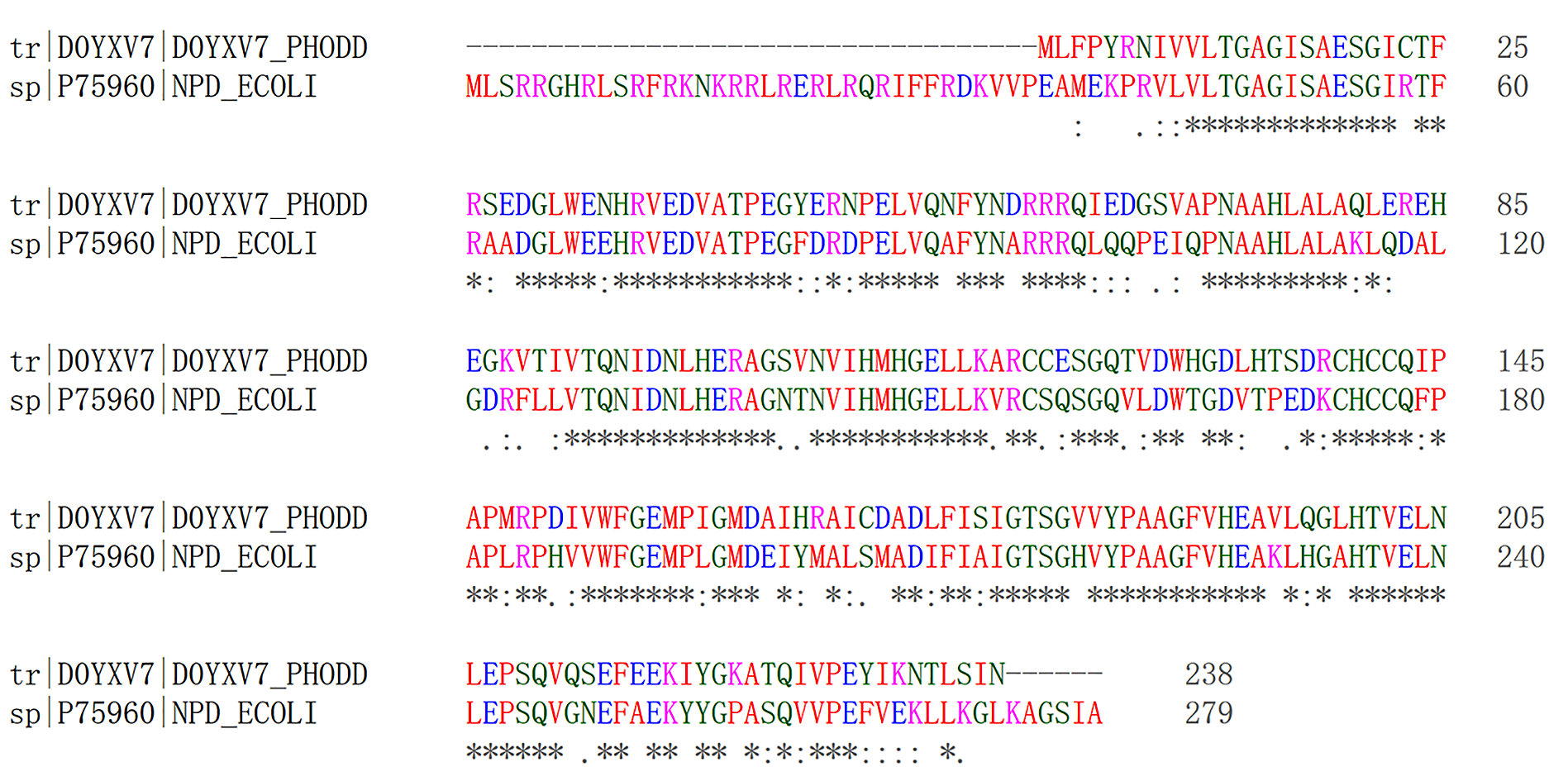
**

**Figure S5. Sequence comparison of CobB in *P. damselae* and *E. coli*.** The sequence alignment of CobB in *P. damselae* and *E. coli* (accession names D0YXV7_PHODD and NPD_ECOLI, respectively) was conducted using the sequences obtained from the Swissprot database and analyzed for alignment through the online tool Clustalo (v.1.2.4). The sequence identity between the two sequences was 68.9%, and the score was 864.

**2. Supplemental Tables**

**Table S1**. The K_lac_-modified peptides and proteins identified in *P. damselae*.

**Table S2**. GO functional annotation of K_lac_-modified proteins in *P. damselae*.

**Table S3**. Distribution of subcellular localization of K_lac_-modified proteins in *P. damselae*.

**Table S4**. GO-based enrichment analysis of K_lac_-modified proteins in *P. damselae*.

**Table S5**. KEGG pathway-based enrichment analysis of K_lac_-modified protein in *P. damselae*.

**Table S6**. Protein domain-based enrichment analysis of K_lac_-modified Proteins in *P. damselae*.

**Table S7**. The PPI network of K_lac_-modified proteins in *P. damselae*.

*Note: all supplemental tables are showed in Excel files (.xlsx)*
